# Label-free imaging and analysis of subcellular parts of a living diatom cylindrotheca sp. using optical diffraction tomography

**DOI:** 10.1016/j.mex.2020.100889

**Published:** 2020-04-23

**Authors:** Kazuo Umemura, Yuji Matsukawa, Yuki Ide, Shigeki Mayama

**Affiliations:** a Biophysics-Section, Department of Physics, Faculty of Science Division II, Tokyo University of Science, 1-3 Kagurazaka, Shinjuku, Tokyo 1628601, Japan; b Faculty of Education, Tokyo Gakugei University, 4-1-1 Nukui-kita-machi, Koganei, Tokyo 184-8511, Japan

**Keywords:** Diatom, Photorefractive indexes (RI), Optical diffraction tomography (ODT), Holotomography (HT), Quantitative phase imaging (QPI), Digital holographic microscope (DHM)

## Abstract

Optical diffraction tomography is an emerging label-free microscopic technique with its capability of label-free, quantitative, and rapid imaging of biological samples. In this work, we present the imaging and analysis of a living diatom *Cylindrotheca* sp. in seawater without using any pretreatment such as fluorescence staining. The 3D refractive index (RI) of a living diatom cell was measured, to which quantitative image analysis was perform to investigate subcellular parts of the diatom. Each part of the cell was well distinguished as RI values and distributions. From the analysis, RI values of frustules, protoplasm, vacuole, and chloroplast were estimated to be in the range of 1.352−1.388, 1.363−1.381, 1.388−1.395, and 1.403−1.436, respectively. Our results suggest that the present method will be a powerful tool not only for observing diatom cells but also for studying various cells and mesoscopic materials.

• Subcellular parts of a living diatom cell was well visualized by digital holographic microscope.
• Subcellular parts could be identified as differences of refractive indexes.
• The observation was achieved without any pre-treatment of the living cell.

## Highlights

• Subcellular parts of a living diatom cell was well visualized by digital holographic microscope.
• Subcellular parts could be identified as differences of refractive indexes.
• The observation was achieved without any pre-treatment of the living cell.

## Graphical abstract

**Figure:**
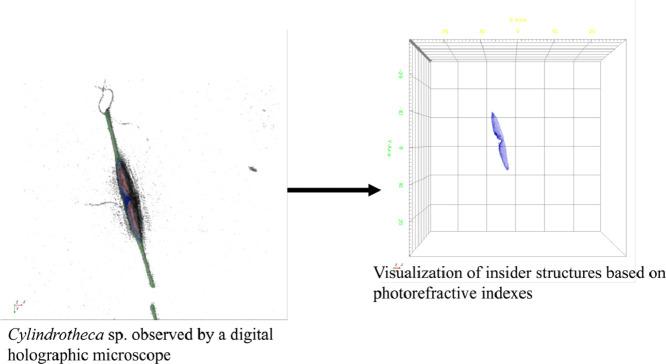

Specifications table

**Table:** 

Subject Area	
More specific subject area	*Microbiological methods*
Method name	*Digital holographic microscope (DHM)*
Name and reference of original method	*N/A*
Resource availability	*N/A*

## Method details

Quantitative phase imaging (QPI) techniques have been exploited to investigate both morphology and optical properties of living cells, due to its label-free and quantitative imaging capability. [1-5] For example, 3D QPI techniques, such as optical diffraction tomography (ODT) or holotomography (HT), reconstructs three-dimensional structures of a living cell from multiple 2D optical field images with various illumination angles. The reconstructed 3D refractive index (RI) distributions of a cell can provide both the morphological information about the cell and the quantification of the dry mass of the cell and the dry mass concentration. [6-10]

Although QPI provides several attractive benefits in bioimaging - without any pre-treatments such as fixation of cells or staining with dyes, its applications to the field of biology and medicine as a standard method has been stymied due to the limitations of instruments, including limited spatial resolution and unfavorable usability in biological laboratories. For the last decade, there have been significant developments in the instrumentations of QPI. [2,7,11-17] For example, the spatial and temporal resolution of QPI had been improved and reached the limitations of far-field diffraction resolution and the speed of a high-speed image sensor. [18-22] Recently, many researchers have intensively reported biological applications of QPI, in particular, medical applications. For example, Moon et al. reported the observation of rhythm strip and parameters of synchronization of human-induced pluripotent stem cell (iPS). [23,24] Mugunano et al. evaluated cadmium-induced cell apoptosis. [25] Ugele et al. quantitatively compared differentiation of leukocytes (DIFF) with eight leukocyte subtypes. [4] Stretching cells were one of the unique applications. [26] On the other hand, red blood cells (RBCs) have also been interests for many researchers. [6,27-29] Furthermore, the quantitative imaging capability of QPI and its synergistic applications in combination with artificial intelligence (AI) became one of the hot topics. [30-36]

One of the important but not fully explored applications of QPI is the study of diatoms. Diatoms are major photosynthetic planktons that produce 20% of primary products on the earth. Previously, the shape of diatoms was measured using holographic microscopy [37-39]. However, the spatial resolution had been mostly limited. Recently, Zetsche reported improved QPI images of diatoms in details in 2014 with an enhanced spatial resolution using DHM, but it only addressed 2-D information. [1] More recently, due to the advances in ODT and HT techniques, 3-D RI tomograms of diatoms have been measured. Simon et al. measured the 3D RI tomogram of a non-living *Coscinusdiscus sp.* diatom using ODT. [40] Lee et al. reported the 3D RI tomograms of phytoplanktons, including *Naviculaes, Pseudo-nitzschia, and Thalassiosira*. [41] However, the 3D RI tomogram of living diatoms and their quantitative analysis have not been performed.

In this paper, we report the measurements of 3D RI tomograms of living *cylindrotheca sp.* diatom in seawater using HT. We also perform the quantitative analysis to the measured RI tomograms and systematically investigate the subcellular parts of the diatom, including frustules, protoplasm, vacuole, and chloroplast of a living diatom cell.

Unlabeled live diatom samples were prepared and imaged using HT. The sample preparation is as follows. An aliquot of seawater Katase-Enoshima area was diluted into Guillard f/2 culture medium and incubated for several weeks at 20 ℃. Then, an appropriate volume of the incubated seawater was injected in an imaging dish (TomoDish, Tomocube Inc., Daejeon, South Korea), and then, directly observed using an HT instrument (HT-2, Tomocube Inc., Daejeon, South Korea). For the observation, an objective lens was immersed in water, not in a culture medium. The theoretical spatial resolution of the used HT system is 110 nm and 360 nm for lateral and axial direction, respectively.

In order to validate the HT system, we measured latex beards (6 μm in diameter, 19102-2, Polysciences, Inc., Warrington, PA) as a standard sample. The representative RI tomograms of the latex beads are presented in Supplementary Information Fig. S1. The averaged RI value of the beads was measured as 1.594 ± 0.001 (*n* = 61). The value is well corresponded to the RI values of the beads provided by the manufacturer (1.5983).

**Figure 1:**
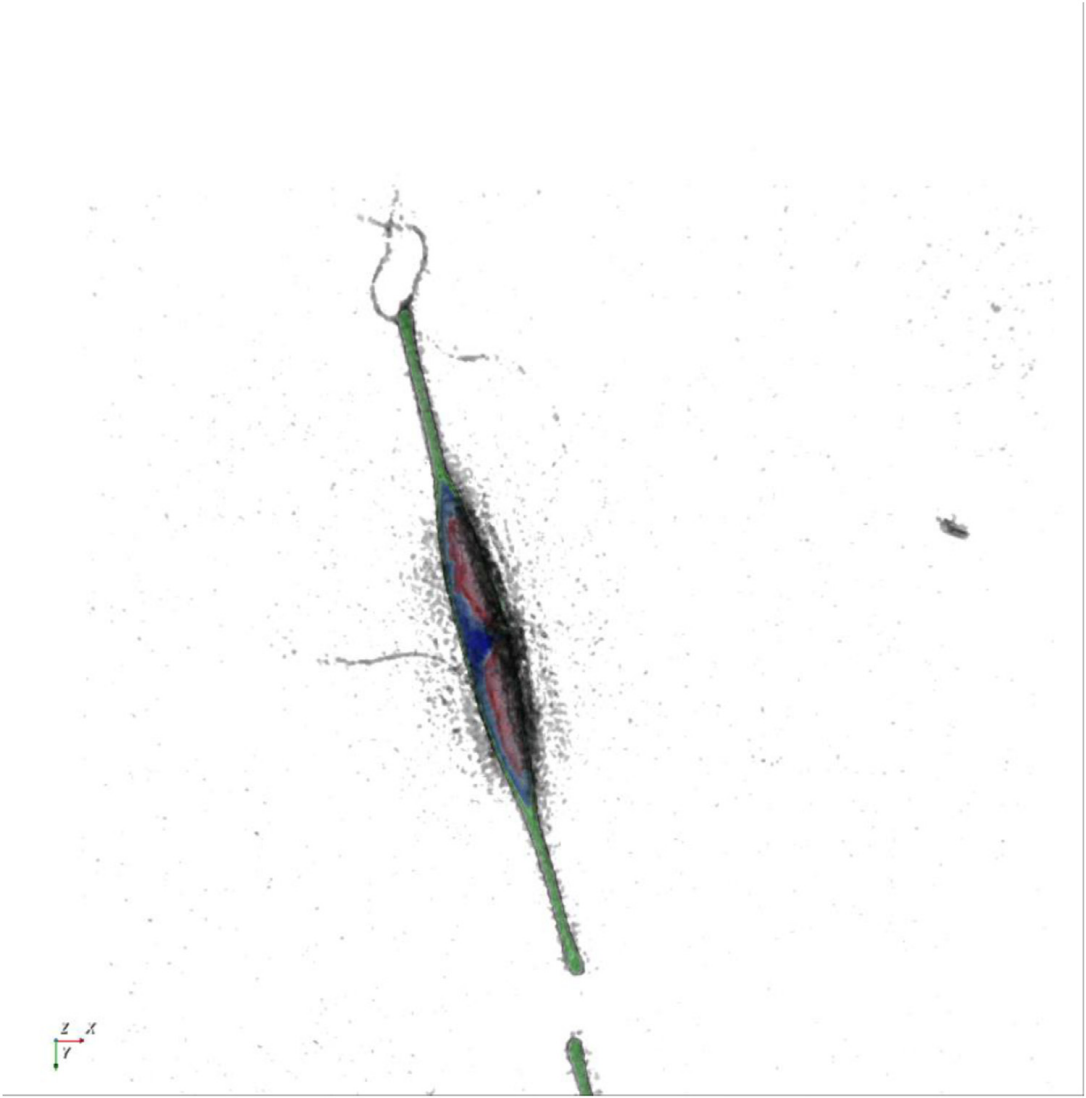
A RI tomogram of a living *cylindrotheca* sp. cell. The lateral field of view was 58.6 μm × 58.6 μm. The color represents RI values.

Figure 1 shows a representative RI tomogram image of a diatom cell, *cylindrotheca* sp. The unique morphology of the cell was well observed. In addition, because an aliquot of seawater was directly observed, unknown fiber-like objects were also clearly observed.

**Figure 2:**
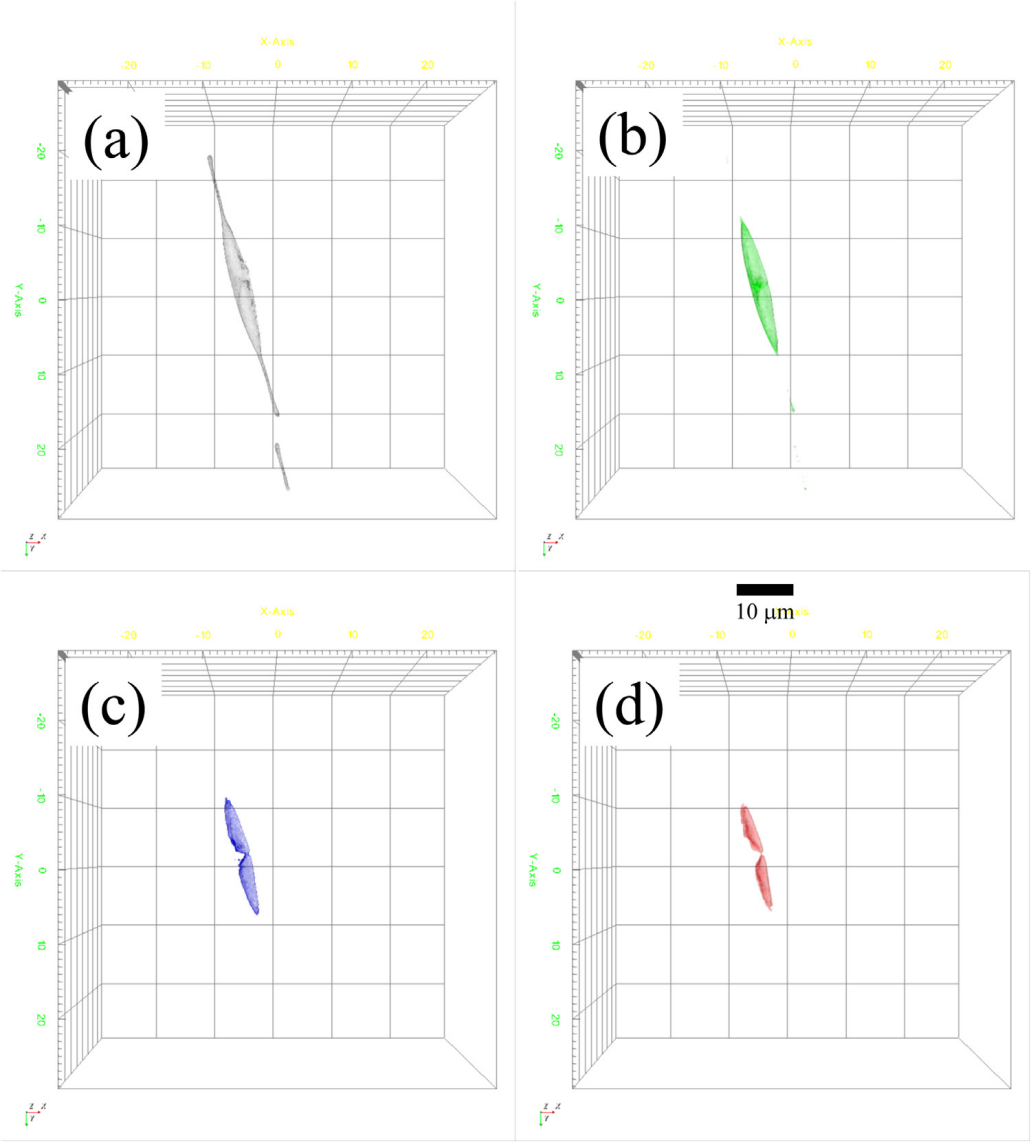
The subparts of the living *cylindrotheca* sp. cell separated by their RI values. (a) Black. RI range: 1.352 to 1.357. (b) Green. RI range: 1.363 to 1.381. (c) Blue. RI range 1.388 to 1.395. (d) Red. RI range: 1.403 to 1.436. The lateral field of view was 58.6 μm × 58.6 μm.

In order to investigate the internal structures of the diatom cell, the measured RI tomogram was analyzed based on their spatial distributions and RI values. Figure 2 represents four different 3D structures of the cell. From the measured RI tomogram, four different 3D images were retrieved based on specific ranges of the RI values. The black volume in Fig. 2(a) represent cellular parts that had RI values between 1.352 and 1.357. For visualization purpose, the extracted volumes in Fig. 2 are also presented as video clips (see Supplementary Information Fig. S2). The morphology of the black volume is almost similar to the well-known frustule structures of *cylindrotheca* sp. [42,43] This means, diatom frustule morphology could be visualized by observing RI distribution.

The green, blue, and red volumes in Figs. 2(b)−(d) were generated with the RI ranges 1.363−1.381, 1.388−1.395, and 1.403−1.436. The green, blue, and red structures could be understood as protoplasm, vacuole, and chloroplast based on their morphologies. In the case of the red parts, ‘volume’ and ‘volume of color’ in the table indicated almost the same values. On the other hand, in blue parts, ‘volume’ value was much larger than ‘volume color’ value. If we think red and blue parts revealed vacuole and chloroplast, the difference is reasonable for the following reason. The chloroplast is totally chloroplast, so that RI values might be uniform. That is why ‘volume’ and ‘volume of color’ showed similar values. Vacuole membrane and vacuole inside might have different RI values. So, when the vacuole membrane is illuminated, inside of vacuole might be not illuminated. Although further experiments are necessary, we think the four volumes extracted with their RI values are reasonable to distinguish each part of the cell structures. Time-lapse observation of dynamics of living cells are probably possible with this method. On the other hand, there are not enough theoretical explanation for the above threshold RI values at this moment. Thus, we observed a well-known diatom cell in this work. Accumulation of observation data of various types of cells will be expected to establish the new method.

Table 1.

**Table 1:** Assignment of frustules (black), protoplasm (green), vacuole (blue), and chloroplast (red) based on RI analysis.

	Black	Green	Blue	Red
Min RI	1.3520	1.3630	1.3880	1.4030
Max RI	1.3570	1.3810	1.3950	1.4360
Volume (um³)	147.4674	109.0293	63.3513	42.5344
Volume of color (um³)	38.4381	45.6780	20.8169	42.5344
Surface area (um²)	288.5568	197.5795	145.0073	114.9186
Projected area (um²)	57.2084	43.5051	33.1990	26.5573
Mean RI	1.3878	1.3989	1.4164	1.4271
Concentration (pg/um³)	0.2849	0.3432	0.4351	0.4917
Dry mass (pg)	42.0068	37.4194	27.5611	20.9152
Dry mass of color (pg)	4.5874	9.8583	6.6459	20.9152
Sphericity	0.4678	0.5586	0.5300	0.5128
Threshold RI	1.3520	1.3630	1.3880	1.4030

Conclusions

Frustules, protoplasm, vacuole, and chloroplast of a living diatom cell were well distinguished based on the HT observation without any staining and isolation. The RI values of each part were also estimated from the measured RI tomograms. Our results clearly revealed future potentials of HT method in researches of diatom and other microorganisms.

Acknowledgments

This research was partially supported by Research Grants of Kitano Foundation of Lifelong Integrated Education.

**Acknowledgements:**

This research was partially supported by Research Grants of Kitano Foundation of Lifelong Integrated Education.

**Conflict of interest statement:**

The authors have no conflicts of interest directly relevant to the content of this article.

**References:**

[1] C.B. Field, M.J. Behrenfeld, J.T. Randerson, P. Falkowski, Primary production of the biosphere: Integrating terrestrial and oceanic components, Science 281 (1998) 237-240. 10.1126/science.281.5374.237.

[2] X.Y. Quan, M. Kumar, O. Matoba, Y. Awatsuji, Y. Hayasaki, S. Hasegawa, H. Wake, Three-dimensional stimulation and imaging-based functional optical microscopy of biological cells, Opt. Lett. 43 (2018) 5447-5450. 10.1364/ol.43.005447.

[3] P. Stepien, D. Korbuszewski, M. Kujawinska, Digital Holographic Microscopy with extended field of view using tool for generic image stitching, Etri J. 41 (2019) 73-83. 10.4218/etrij.2018-0499.

[4] M. Ugele, M. Weniger, M. Stanzel, M. Bassler, S.W. Krause, O. Friedrich, O. Hayden, L. Richter, Label-Free High-Throughput Leukemia Detection by Holographic Microscopy, Adv. Sci. 5 (2018) 9 1800761. 10.1002/advs.201800761.

[5] Y. Park, C. Depeursinge, G. Popescu, Quantitative phase imaging in biomedicine, Nature Photonics 12 (2018) 578.

[6] A. Brodoline, N. Rawat, D. Alexandre, N. Cubedo, M. Gross, 4D compressive sensing holographic microscopy imaging of small moving objects, Opt. Lett. 44 (2019) 2827-2830. 10.1364/ol.44.002827.

[7] F. Dubois, N. Callens, C. Yourassowsky, M. Hoyos, P. Kurowski, O. Monnom, Digital holographic microscopy with reduced spatial coherence for three-dimensional particle flow analysis, Appl. Optics 45 (2006) 864-871. 10.1364/ao.45.000864.

[8] K. Jaferzadeh, I. Moon, M. Bardyn, M. Prudent, J.D. Tissot, B. Rappaz, B. Javidi, G. Turcatti, P. Marquet, Quantification of stored red blood cell fluctuations by time-lapse holographic cell imaging, Biomed. Opt. Express 9 (2018) 4714-4729. 10.1364/boe.9.004714.

[9] R. Barer, Determination of dry mass, thickness, solid and water concentration in living cells, Nature 172 (1953) 1097.

[10] G. Popescu, Y. Park, N. Lue, C. Best-Popescu, L. Deflores, R.R. Dasari, M.S. Feld, K. Badizadegan, Optical imaging of cell mass and growth dynamics, American Journal of Physiology-Cell Physiology 295 (2008) C538-C544.

[11] D. Dannhauser, D. Rossi, P. Memmolo, A. Finizio, P. Ferraro, P.A. Netti, F. Causa, Biophysical investigation of living monocytes in flow by collaborative coherent imaging techniques, Biomed. Opt. Express 9 (2018) 5194-5204. 10.1364/boe.9.005194.

[12] A. De Angelis, M.A. Ferrara, G. Coppola, L. Di Matteo, L. Siani, B. Dale, G. Coppolad, A.C. De Luca, Combined Raman and polarization sensitive holographic imaging for a multimodal label-free assessment of human sperm function, Sci Rep 9 (2019) 15 4823. 10.1038/s41598-019-41400-0.

[13] R.K. Gupta, M.Z. Chen, G.P.A. Malcolm, N. Hempler, K. Dholakia, S.J. Powis, Label-free optical hemogram of granulocytes enhanced by artificial neural networks, Opt. Express 27 (2019) 13706-13720. 10.1364/oe.27.013706.

[14] E. Makdasi, O. Laskar, E. Milrot, O. Schuster, S. Shmaya, S. Yitzhaki, Whole-Cell Multiparameter Assay for Ricin and Abrin Activity-Based Digital Holographic Microscopy, Toxins 11 (2019) 16 174. 10.3390/toxins11030174.

[15] A.G. Wingren, Moving into a new dimension: Tracking migrating cells with digital holographic cytometry in 3D, Cytom. Part A 95A (2019) 144-146. 10.1002/cyto.a.23679.

[16] W. Xiao, Q.X. Wang, F. Pan, R.Y. Cao, X.T. Wu, L.W. Sun, Adaptive frequency filtering based on convolutional neural networks in off-axis digital holographic microscopy, Biomed. Opt. Express 10 (2019) 1613-1626. 10.1364/boe.10.001613.

[17] Z.M. Yang, Z.J. Liu, W.L. He, J.T. Dou, X. Liu, C. Zuo, Automatic high order aberrations correction for digital holographic microscopy based on orthonormal polynomials fitting over irregular shaped aperture, J. Opt. 21 (2019) 9 045609. 10.1088/2040-8986/ab0e63.

[18] Y. Cotte, F. Toy, P. Jourdain, N. Pavillon, D. Boss, P. Magistretti, P. Marquet, C. Depeursinge, Marker-free phase nanoscopy, Nature Photonics 7 (2013) 113.

[19] K. Kim, K.S. Kim, H. Park, J.C. Ye, Y. Park, Real-time visualization of 3-D dynamic microscopic objects using optical diffraction tomography, Opt. Express 21 (2013) 32269-32278.

[20] C. Allier, L. Herve, O. Mandula, P. Blandin, Y. Usson, J. Savatier, S. Monneret, S. Morales, Quantitative phase imaging of adherent mammalian cells: a comparative study, Biomed. Opt. Express 10 (2019) 2768-2783. 10.1364/boe.10.002768.

[21] X. Fan, J.J. Healy, K. O'Dwyer, B.M. Hennelly, Label-free color staining of quantitative phase images of biological cells by simulated Rheinberg illumination, Appl. Optics 58 (2019) 3104-3114. 10.1364/ao.58.003104.

[22] H. Funamizu, Y. Aizu, Three-dimensional quantitative phase imaging of blood coagulation structures by optical projection tomography in flow cytometry using digital holographic microscopy, J. Biomed. Opt. 24 (2019) 6 031012. 10.1117/1.jbo.24.3.031012.

[23] I. Moon, E. Ahmadzadeh, K. Jaferzadeh, N. Kim, Automated quantification study of human cardiomyocyte synchronization using holographic imaging, Biomed. Opt. Express 10 (2019) 610-621. 10.1364/boe.10.000610.

[24] I. Moon, K. Jaferzadeh, E. Ahmadzadeh, B. Javidi, Automated quantitative analysis of multiple cardiomyocytes at the single-cell level with three-dimensional holographic imaging informatics, J. Biophotonics 11 (2018) 12 UNSP e201800116. 10.1002/jbio.201800116.

[25] M. Mugnano, P. Memmolo, L. Miccio, S. Grilli, F. Merola, A. Calabuig, A. Bramanti, E. Mazzon, P. Ferraro, In vitro cytotoxicity evaluation of cadmium by label-free holographic microscopy, J. Biophotonics 11 (2018) 8 UNSP e201800099. 10.1002/jbio.201800099.

[26] S. Yadav, R. Vadivelu, M. Ahmed, M. Barton, N.T. Nguyen, Stretching cells - An approach for early cancer diagnosis, Exp. Cell Res. 378 (2019) 191-197. 10.1016/j.yexcr.2019.01.029.

[27] Y. Fang, N.M. Yu, Y.Q. Jiang, Super-Resolution Lensless Imaging of Cells Using Brownian Motion, Appl. Sci.-Basel 9 (2019) 11 2080. 10.3390/app9102080.

[28] K. Lee, S. Shin, Z. Yagoob, P.T.C. So, Y. Park, Low-coherent optical diffraction tomography by angle-scanning illumination, J. Biophotonics 12 (2019) 10 UNSP e201800289. 10.1002/jbio.201800289.

[29] J.Q. Liu, L.Q. Zhu, F. Zhang, M.L. Dong, X.H. Qui, Microdeformation of RBCs under oxidative stress measured by digital holographic microscopy and optical tweezers, Appl. Optics 58 (2019) 4042-4046. 10.1364/ao.58.004042.

[30] H. Byeon, T. Go, S.J. Lee, Deep learning-based digital in-line holographic microscopy for high resolution with extended field of view, Opt. Laser Technol. 113 (2019) 77-86. 10.1016/j.optlastec.2018.12.014.

[31] T. Go, G.Y. Yoon, S.J. Lee, Learning-based automatic sensing and size classification of microparticles using smartphone holographic microscopy, Analyst 144 (2019) 1751-1760. 10.1039/c8an02157k.

[32] S.J. Kim, C.Q. Wang, B. Zhao, H. Im, J. Min, H.J. Choi, J. Tadros, N.R. Choi, C.M. Castro, R. Weissleder, H. Lee, K.M. Lee, Deep transfer learning-based hologram classification for molecular diagnostics, Sci Rep 8 (2018) 12 17003. 10.1038/s41598-018-35274-x.

[33] C. Trujillo, J. Garcia-Sucerquia, Automatic detection and counting of phase objects in raw holograms of digital holographic microscopy via deep learning, Opt. Lasers Eng. 120 (2019) 13-20. 10.1016/j.optlaseng.2019.02.010.

[34] Y. Jo, H. Cho, S.Y. Lee, G. Choi, G. Kim, H.-s. Min, Y. Park, Quantitative phase imaging and artificial intelligence: a review, IEEE Journal of Selected Topics in Quantum Electronics 25 (2018) 1-14.

[35] A. Mahjoubfar, C.L. Chen, B. Jalali, Artificial Intelligence in Label-free Microscopy, Springer2017.

[36] Y. Jo, S. Park, J. Jung, J. Yoon, H. Joo, M.-h. Kim, S.-J. Kang, M.C. Choi, S.Y. Lee, Y. Park, Holographic deep learning for rapid optical screening of anthrax spores, Science advances 3 (2017) e1700606.

[37] B.D.R.D. ALMEIDA S.P., CAIRNS J.JR.DICKSON K.L., LANZA G.R., Holographic microscopy of diatoms, Transactions of the Kansas Academy of Science (1903)∼ 74 (1971) 257-260.

[38] J. Cairns, S.P. Almeida, H. Fujii, Automated Identification Of Diatoms, Bioscience 32 (1982) 98-102. 10.2307/1308561.

[39] J. Cairns, K.L. Dickson, J. Slocomb, ABCs Of Diatom Identification Using Laser Holography, Hydrobiologia 54 (1977) 7-16. 10.1007/bf00018766.

[40] M. Debailleul, B. Simon, V. Georges, O. Haeberlé, V. Lauer, Holographic microscopy and diffractive microtomography of transparent samples, Measurement Science and Technology 19 (2008) 074009.

[41] S. Lee, K. Kim, A. Mubarok, A. Panduwirawan, K. Lee, S. Lee, H. Park, Y. Park, High-resolution 3-D refractive index tomography and 2-D synthetic aperture imaging of live phytoplankton, Journal of the Optical Society of Korea 18 (2014) 691-697.

[42] S. Balzano, I. Percopo, R. Siano, P. Gourvil, M. Chanoine, D. Marie, D. Vaulot, D. Sarno, MORPHOLOGICAL AND GENETIC DIVERSITY OF BEAUFORT SEA DIATOMS WITH HIGH CONTRIBUTIONS FROM THE CHAETOCEROS NEOGRACILIS SPECIES COMPLEX, J. Phycol. 53 (2017) 161-187. 10.1111/jpy.12489.

[43] M. Hildebrand, S. Kim, D. Shi, K. Scott, S. Subramaniam, 3D imaging of diatoms with ion-abrasion scanning electron microscopy, J. Struct. Biol. 166 (2009) 316-328. 10.1016/j.jsb.2009.02.014.

## Appendix Supplementary materials

Image, application 1

