# Supplementary figures and images for: Label-free imaging and analysis of subcellular parts of a living diatom cylindrotheca sp. using optical diffraction tomography
Source: MethodsX. 2020 Apr 23;7:100889. doi: 10.1016/j.mex.2020.100889 (PMC7199005; doi:10.1016/j.mex.2020.100889)

## Slide 1
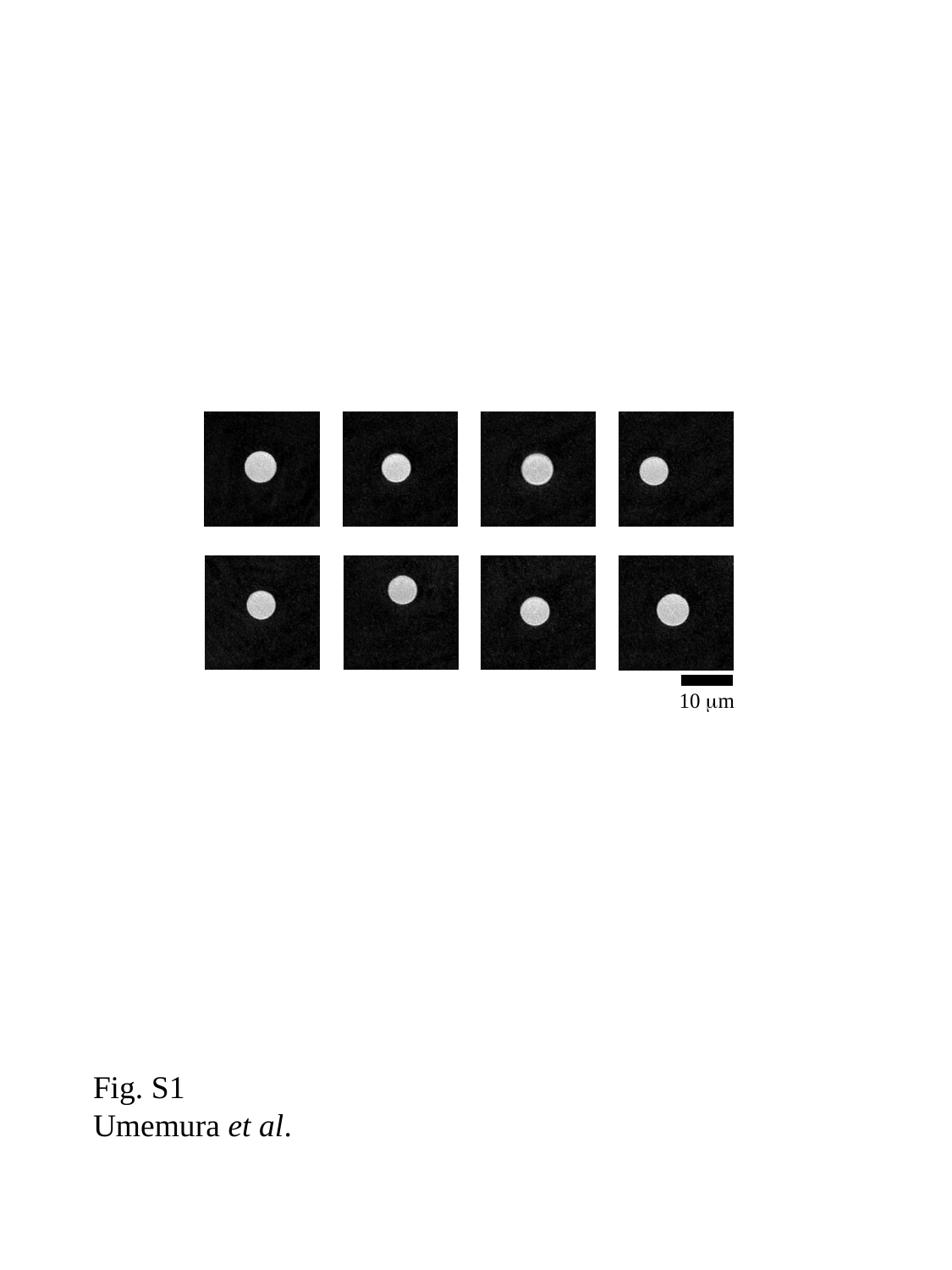

10 mm
Fig. S1
Umemura et al.

## Slide 2
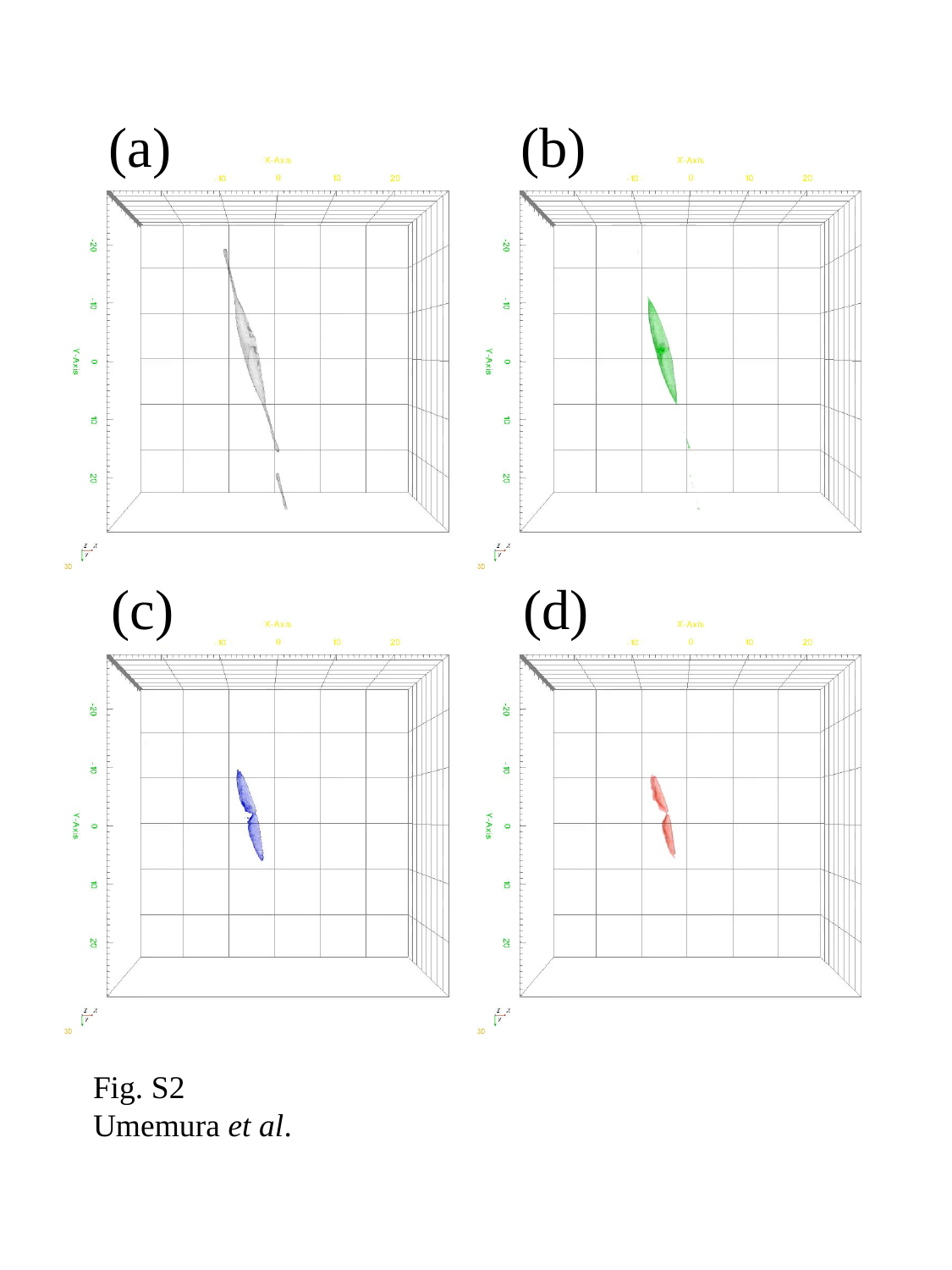

(a)
(b)
(c)
(d)
Fig. S2
Umemura et al.

Supplement: Supplementary file 1 [file mmc1.pptx]
